# Mr. Knowles' Reply to a Norfolk Practitioner

**Published:** 1812-01

**Authors:** E. L. Knowles

**Affiliations:** Soham


### *To the Editors of the Medical and Physical Journal.*

GENTLEMEN,

IN perusing your last Journal, I observed a paper signed a Norfolk Practitioner, on the cases which I have at various times published in your miscellaneous work; in answer to which, I shall lay before your numerous correspondents, a vindication of my conduct, with some observations upon the paper in question. I shall solicit permission to ask this practitioner, if, in the case of diarrhœa, there had been any derangement in the functions of the alimentary canal; must it not have been productive of some of those symptoms which characterise dyspepsia, or some other affection of the stomach, which certainly was not the case; or, if irregularity in the secreting hepatic organs, obstructions in any of the ducts preventing the bile from passing into the intestines, for the assistance of digestion, would it not have been absorbed into the circulating fluids, and produce symptomatic affection, termed Icterus instead of Anasarca? Every person who understands the laws of the animal œconomy, must I think coincide with me upon this subject. He further says that my account of the case is extremely defective; it probably may be so, but I cannot unite with him, that it is defective, because I did not notice the stools; nor do I agree with him that the cause originated from what he specifies, consequently taking notice of the excrements appeared to me to be a matter of no importance, nor do I consider that the quotation from that respectable and learned practitioner, (Mr. Abernethy) is at all applicable in this case. He further says, had he given small alterative doses of mercury, in all probability he would not have experienced the long and tedious trial which he did. Does this gentleman conceive that it is possible for this remedy to have such salutary effects in this case, where the excitement in the bowels required to be allayed, instead of augmented; and does he want to produce a discharge from the salivary glands where there was so much debility existing, and has he ever seen mercury given in diarrhœa? I think he is wandering from the subject, and means dysenteria; it is even improper in this if not joined with opium. Will not stimulus ultimately produce languor?—His observation therefore does not at all convince me that I was proceeding upon erroneous grounds, and that I was not justified in removing the cause by administering a gentle aperient; and decreasing the excitement and peristaltic action by opium, &c. as is mentioned in my letter. I did not pretend to say it was a new new discovery, my only motive in communicating it was for the improvement of the profession; if there was neither novelty or interest, the good intention of it was undeserving criticism?

In April 1810, I wrote a paper, with some observations on Mr. Peck's mode of treating leeches, both before and after suction; wherein I mentioned what the Norfolk Practitioner reflects upon, without making either improvement or interesting remarks, only he has sagaciously told you "he believes there is no insensible membrane upon the mouth of the leech." I certainly made this observation to vindicate my mode of treating those aquatic animals in your former Journal, as I was of opinion that salt would have the same effect there as on any other part of the body, and would be productive of worse consequences, because it would render the animal incapable of supporting life. Does he pretend to say the leech cannot bear pressure, or is salt a proper application after it has been satiated with blood, and can the leech exist longer under the latter than the former mode of treatment?

He further remarks upon the case of Plica Polonica, which was communicated to me, and inserted in this Journal, in May, 1810; the subject of this case was the patient of a surgeon in the neighbourhood of Bury St. Edmunds, by whom the diseased hair was sent to Mr. Rogers, of Newmarket, and likewise the statement of the case which is mentioned in my letter. He further says, "I have seen this Plica, as he is pleased to term it, and there is not the least reason whatever to suppose that it had any thing to do with the disease in question, being simply a morbid affection of the hair, unattended by any of the symptoms which precede or characterise the genuine disease." He likewise accuses me of being neglectful in describing the different symptoms peculiar to this malady. All the information I could obtain respecting the case in question I have published; the only object I had in making it known was its singularity, and not being peculiar to this country. The medical person who saw this case in existence, pronounced it to be a Plica, and so has a professor of anatomy in an English university; and I have since been informed that gentleman has it now in his possession. As this Norfolk Practitioner has denied that there is any similarity between this and a real Plica, I will beg leave to ask him what he means by a morbid growth of the hair, as I am totally unacquainted with any disease of that nature? When a person contradicts he ought to have strong arguments to produce, with a satisfactory elucidation of the disease, but I must confess he is very deficient in that point.

I am extremely sorry to find that my veracity has been called in question respecting a case of Acites, which I published in August, 1810. The subject of this case lived nearly 300 miles from Thetford, (the place where this curious practitioner accuses me of having seen the patient;) in a borough town, in North Wales; and I believe the person who labored under this complaint (but am not positive) is still living. I admit that I have been frequently in the habit of corresponding with Mr. Philip Harrold, but it was when he was only an apprentice, and it was merely a friendly correspondence. I do not recollect his writing to me respecting his sister, neither did I ever see or prescribe for her; all the letters I received from him I was always in the habit of committing to the flames, as I never considered them of sufficient importance to preserve for publication; and, had I so done, should most certainly have acknowledged the author.

This practitioner concludes with observing, that Mr. P. Harrold's letter and my case agree both in circumstances and date; now this case I drew up in 1803, (although I did not publish it until 1810) and he says the letter was communicated to me in 1809. If this practitioner wishes to be further satisfied upon the subject, by writing to me by post, and signing his real name and place of abode, &c. I will give him further information, and permit him to publish it through the medium of your Journal. I assure you gentlemen it would be too painful to my feelings, and too opposite to my nature, to transmit you any thing for publication which had not truth for its basis.

And am, Gentlemen,

Your humble Servant,

E. L. KNOWLES.

*Soham, Nov. 5th,* 1811.

